# Supplementary material for: Trimethoprim: An Old Antibacterial Drug as a Template to Search for New Targets. Synthesis, Biological Activity and Molecular Modeling Study of Novel Trimethoprim Analogs
Source: Molecules. 2019 Dec 27;25(1):116. doi: 10.3390/molecules25010116 (PMC6983048; doi:10.3390/molecules25010116)
Supplement: Supplementary file 1 [file molecules-25-00116-s001.pdf]

Supplementary informations

Trimethoprim: An Old Antibacterial Drug As A Template To Search for New Targets. Synthesis, Biological Activity and Molecular Modeling Study of Novel Trimethoprim Analogues.

Agnieszka Wróbel[a], Dawid Maliszewski[a], Maciej Baradyn[b] and Danuta Drozdowska [a]*

[a]Department of Organic Chemistry, Medical University of Bialystok, Poland;

[b]Department of Theoretical Chemistry, Institute of Chemistry, University of Bialystok, Poland

**Please address all correspondence to: Department of Organic Chemistry, Medical University, Mickiewicza 2A Str., 15-222 Białystok, Poland; e-mail: agnieszkawrobel9@gmail.com.*

Compound 1

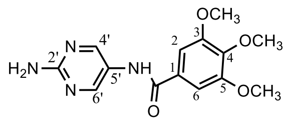

^{1}H NMR

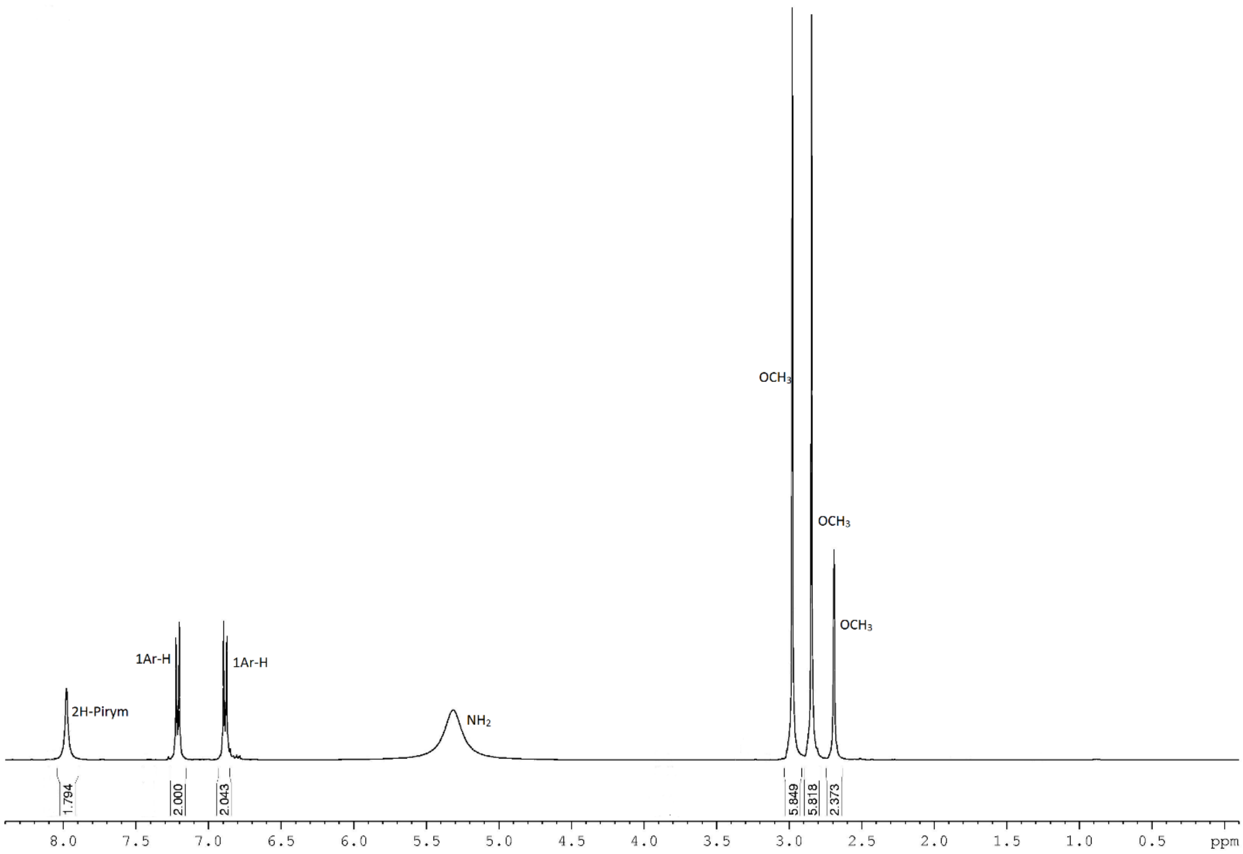

^{13}C NMR

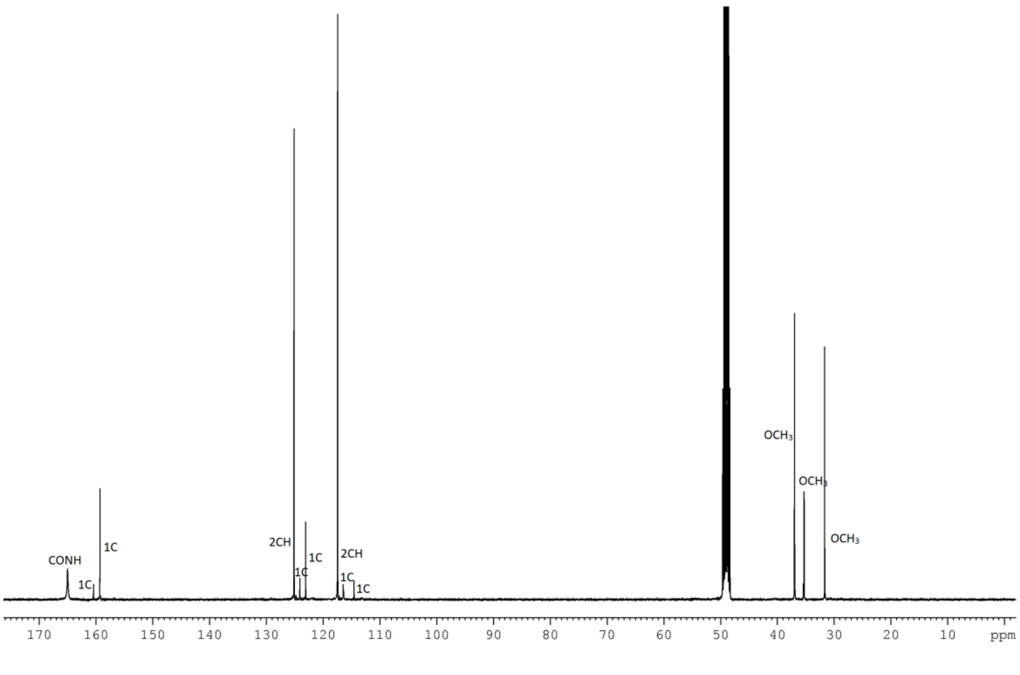

Compound 2

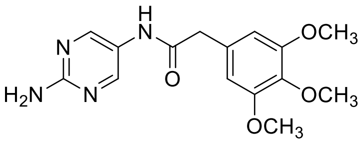

^{1}H NMR

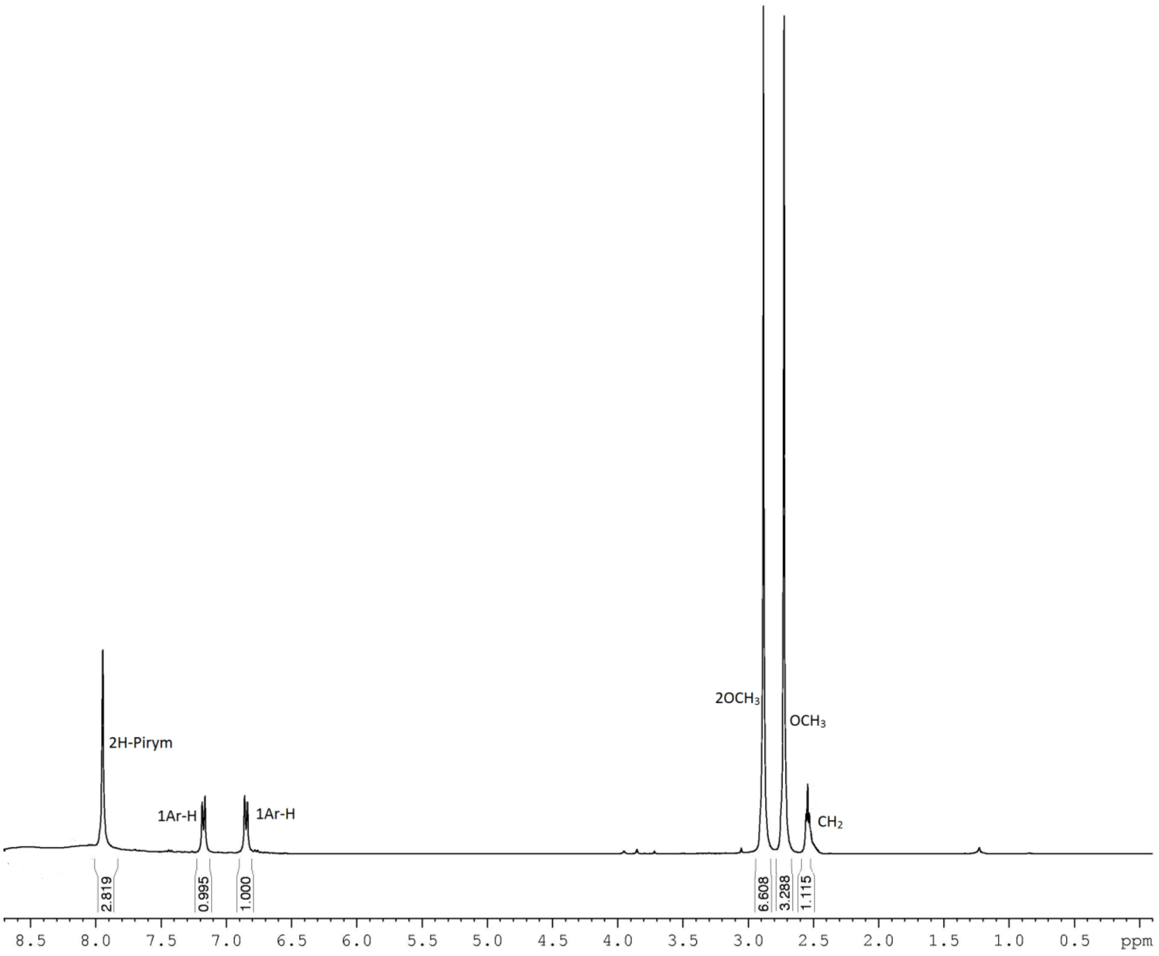

^{13}C NMR

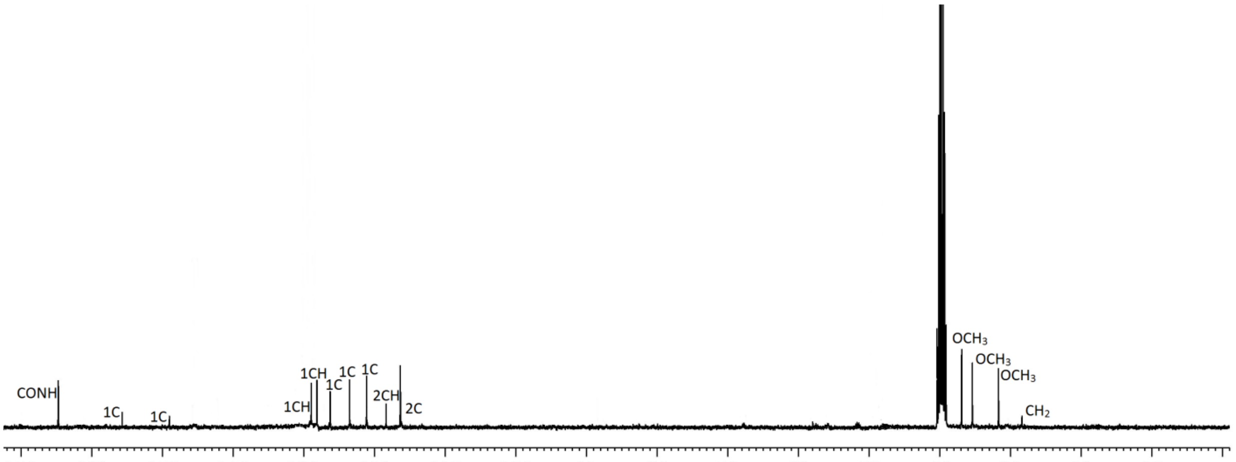

Compound 3

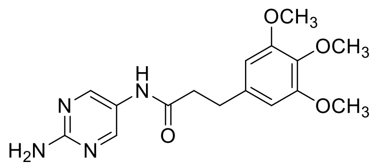

^{1}H NMR

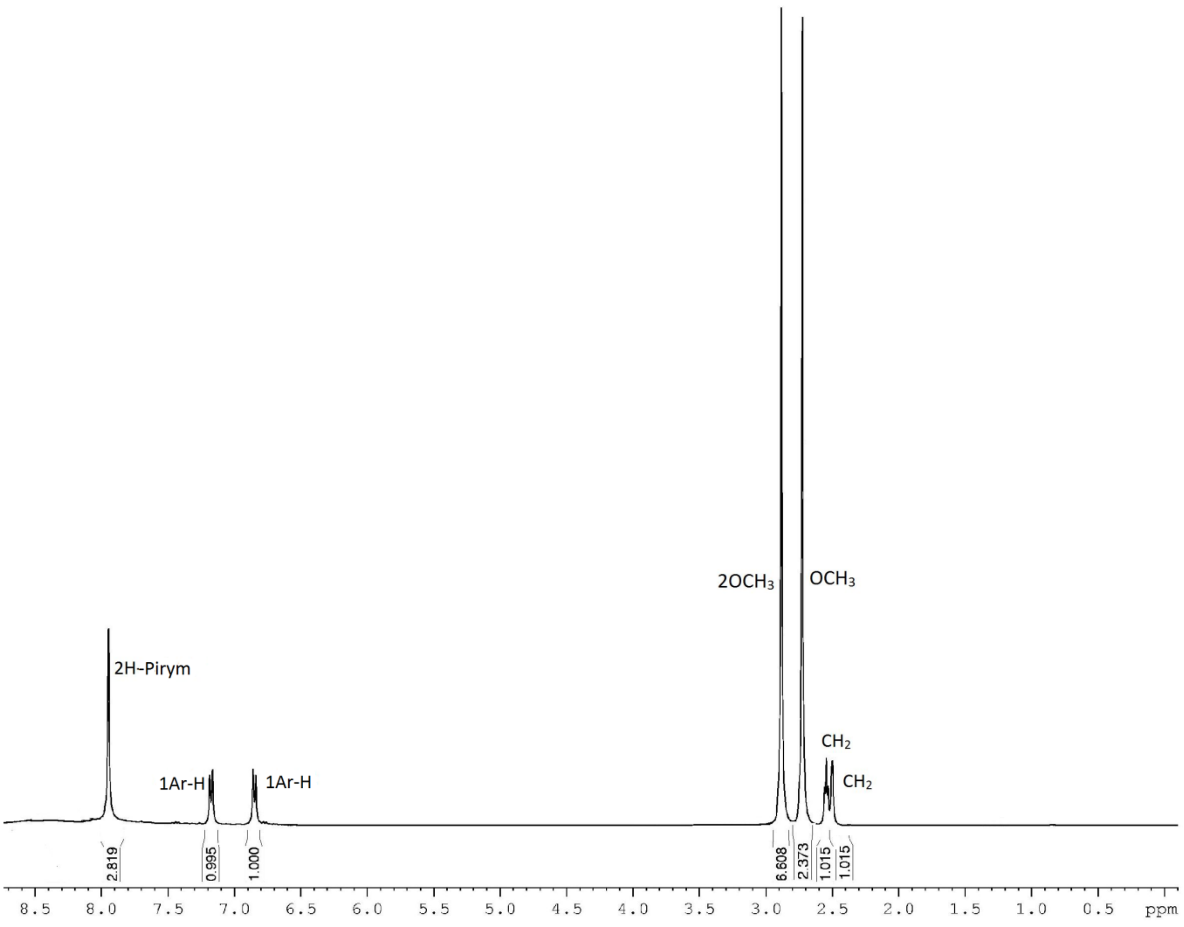

^{13}C NMR

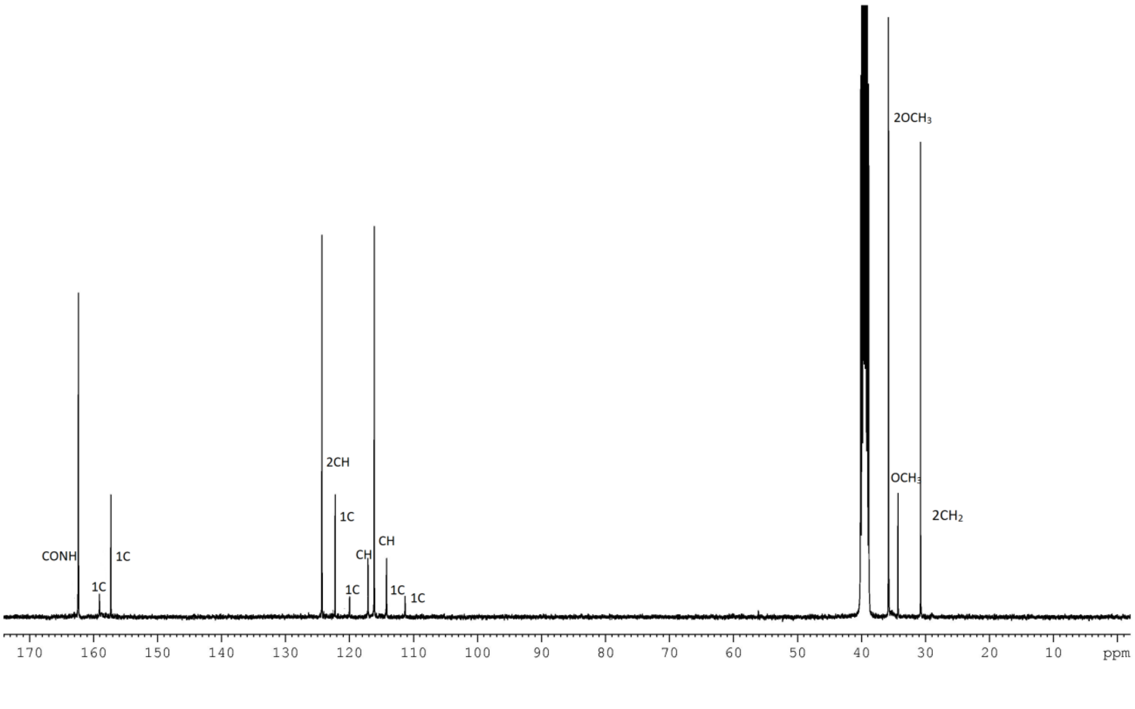

Compound 4

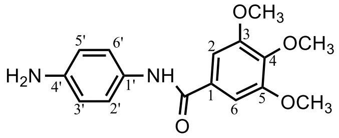

^{1}H NMR ($CDCl_3$)

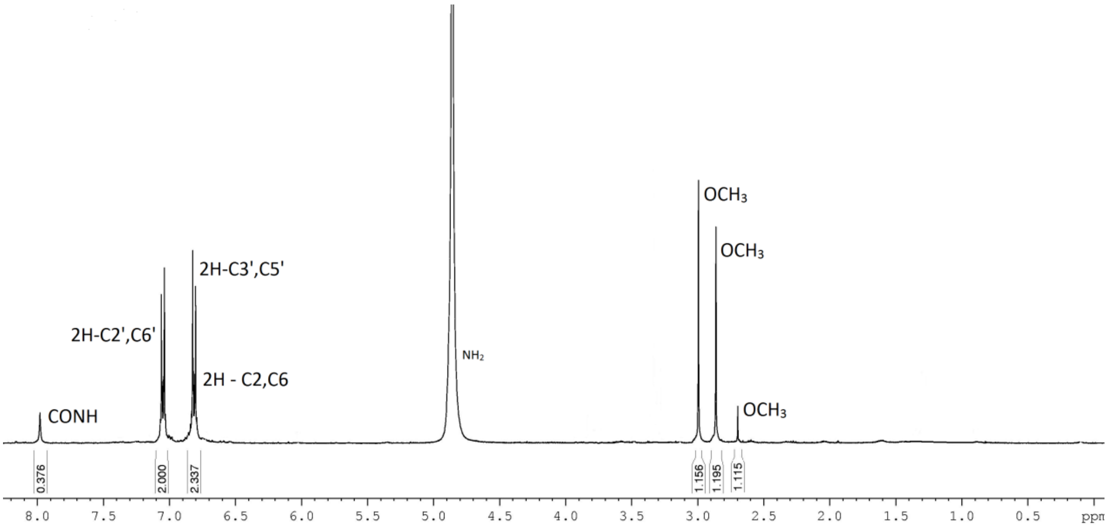

^{13}C NMR NH_2

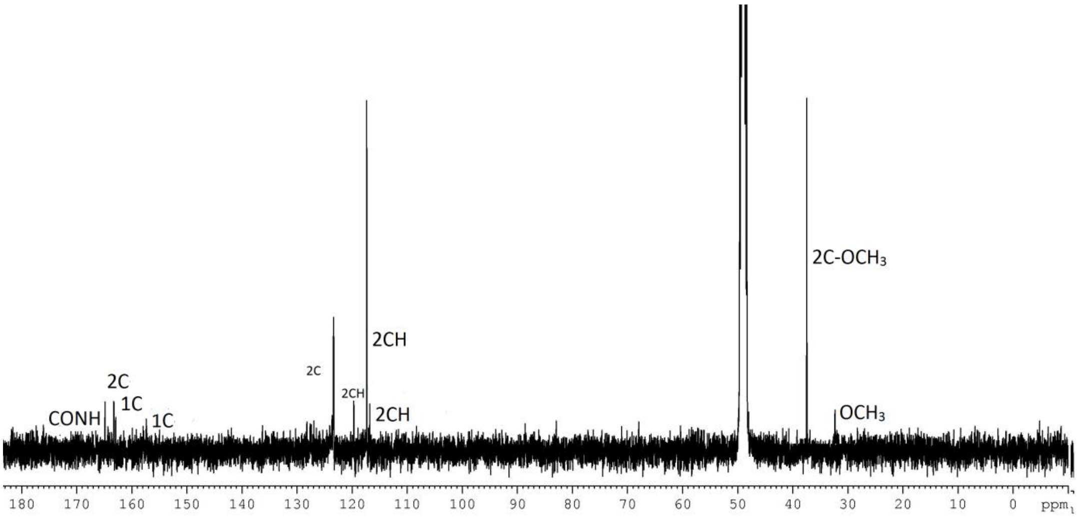

Compound 5

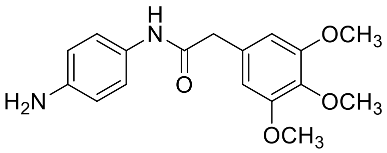

^1H NMR

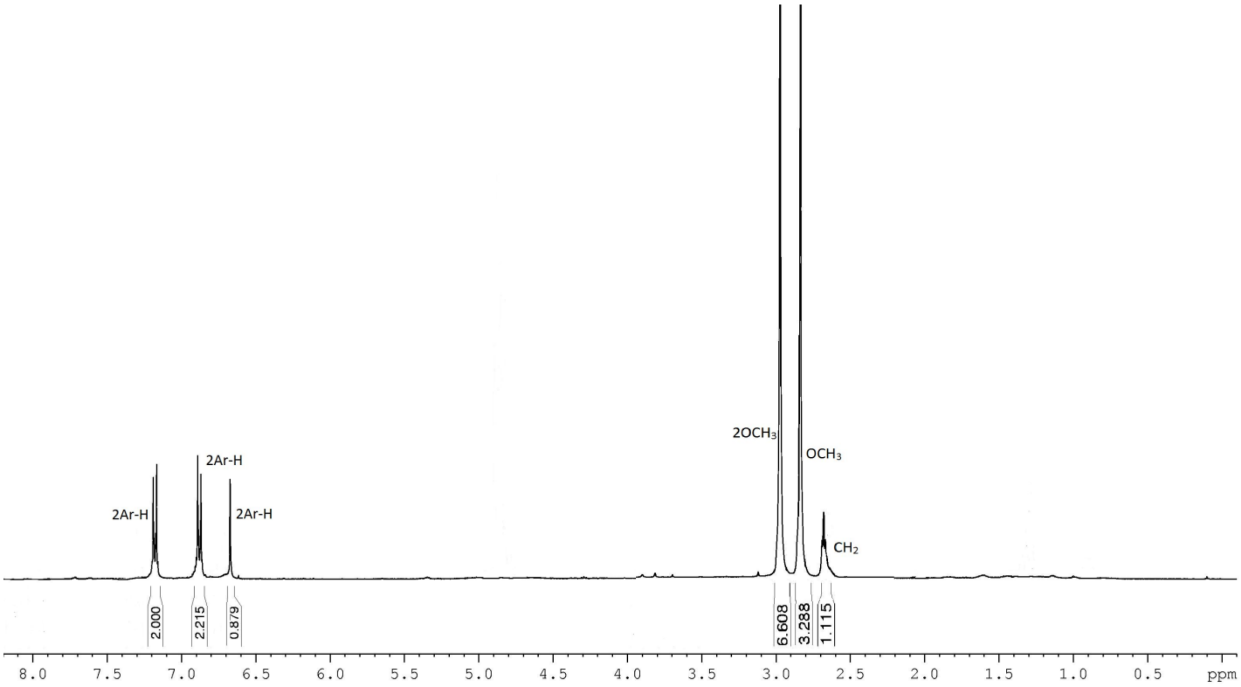

^{13}C NMR

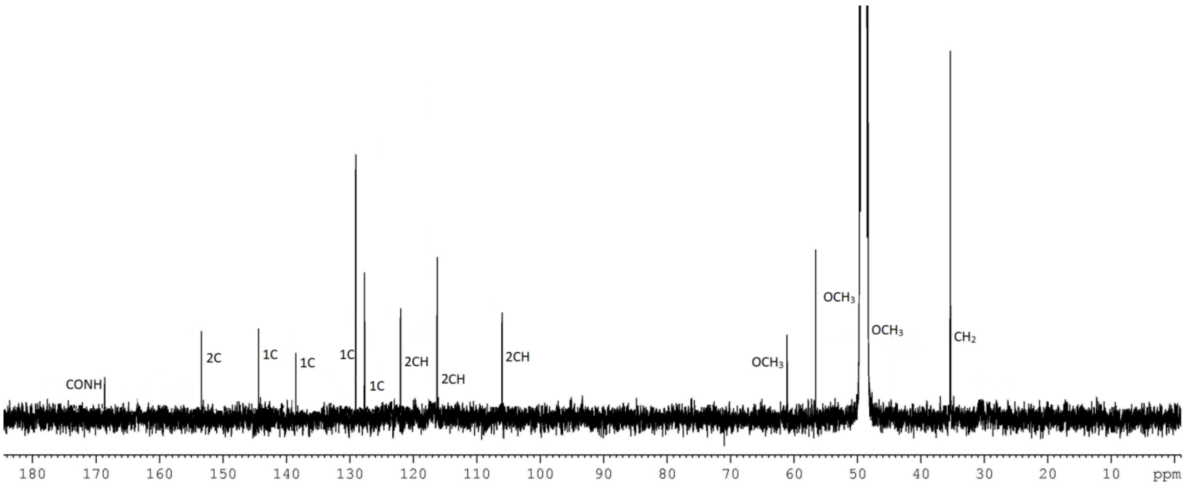

Compound 6

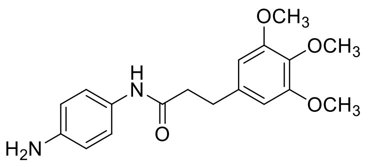

^{1}H NMR

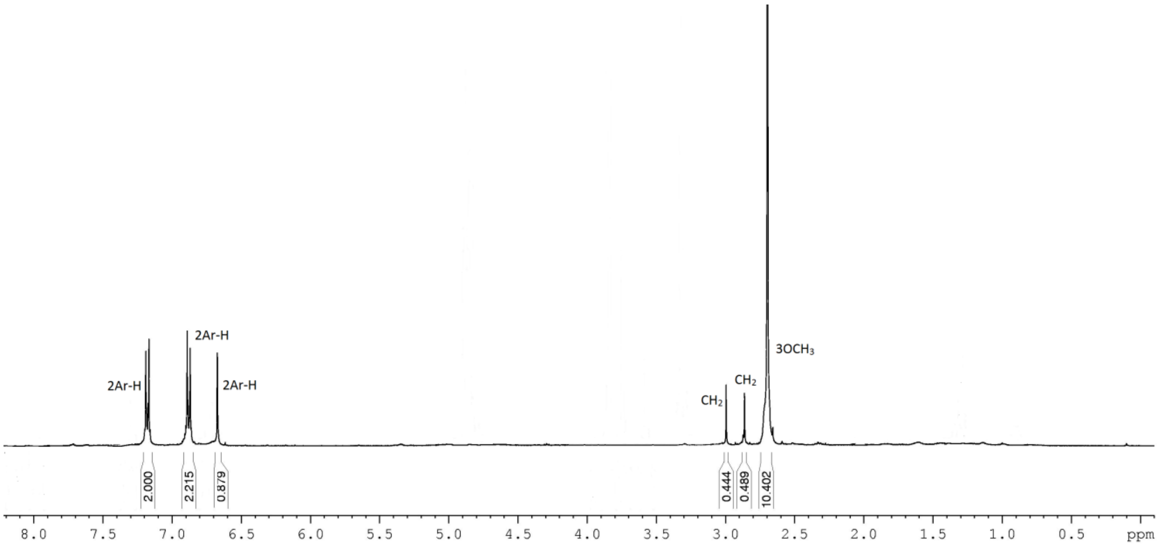

^{13}C NMR

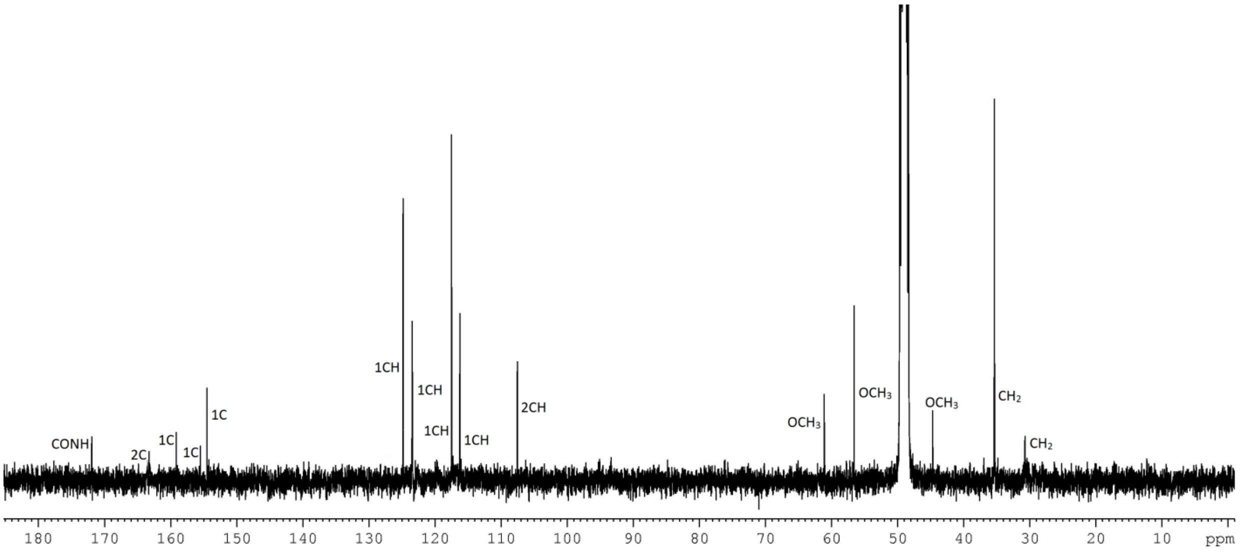

Table SI. List of binding modes of the molecule 2 from this study, as well as RMSD values relative to the best binding mode. Mode 3 is analogous to the binding mode of compound 1 but shows significantly smaller affinity than mode 1 (reversed structure).

mode	affinity (kcal/mol)	dist from best mode	
		rmsd l.b.	rmsd u.b.
1	-8.3	0.000	0.000
2	-8.0	1.162	2.262
3	-7.4	4.123	8.238
4	-7.3	2.794	4.136
5	-7.3	3.938	7.833
6	-7.3	1.983	3.411
7	-7.2	1.548	1.642
8	-7.2	2.752	3.652
9	-7.2	4.258	7.744

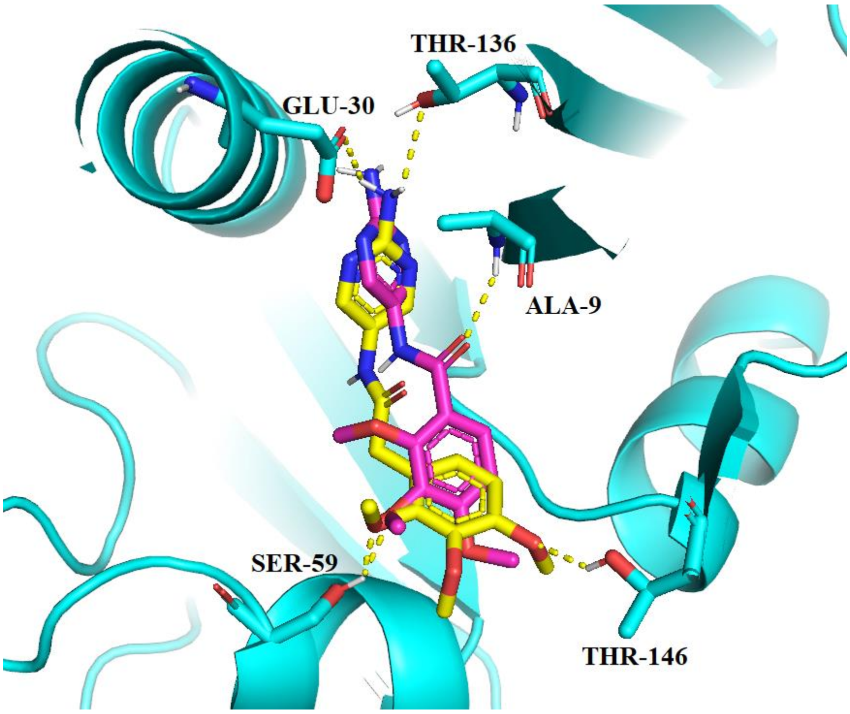

Figure SI. Binding mode comparison of molecule 1 (pink, -7.7 kcal/mol) and mode 3 of molecule 2 (yellow, -7.4 kcal/mol).
